# SiN_x_/SiO_2_-Based Fabry–Perot Interferometer on Sapphire for Near-UV Optical Gas Sensing of Formaldehyde in Air

**DOI:** 10.3390/s24113597

**Published:** 2024-06-03

**Authors:** Reinoud Wolffenbuttel, Declan Winship, David Bilby, Jaco Visser, Yutao Qin, Yogesh Gianchandani

**Affiliations:** 1 Laboratory for Electronic Instrumentation, Department of Microelectronics, Faculty of Electrical Engineering, Mathematics and Computer Science, Delft University of Technology, 2628 CD Delft, The Netherlands; 2 Center for Wireless Integrated MicroSensing and Systems (WIMS2), University of Michigan, Ann Arbor, MI 48109, USA; dejawin@umich.edu (D.W.); yutaoqin@umich.edu (Y.Q.); yogesh@umich.edu (Y.G.); 3 Department of Electrical Engineering and Computer Science, University of Michigan, Ann Arbor, MI 48109, USA; 4 Research and Advanced Engineering, Ford Motor Company, Dearborn, MI 48121, USA; dbilby@ford.com (D.B.); jvisser@ford.com (J.V.)

**Keywords:** optical MEMS, nitride-rich SiN*_x_*, microspectrometer, absorption spectroscopy, CMOS-compatible optical sensors, near-UV optical sensor, optical gas sensor, Bragg reflector with lossy layers, Fabry–Perot resonator with lossy layers

## Abstract

Fabry–Perot interferometers (FPIs), comprising foundry-compatible dielectric thin films on sapphire wafer substrates, were investigated for possible use in chemical sensing. Specifically, structures comprising two vertically stacked distributed Bragg reflectors (DBRs), with the lower DBR between a sapphire substrate and a silicon-oxide (SiO_2_) resonator layer and the other DBR on top of this resonator layer, were investigated for operation in the near-ultraviolet (near-UV) range. The DBRs are composed of a stack of nitride-rich silicon-nitride (SiN*_x_*) layers for the higher index and SiO_2_ layers for the lower index. An exemplary application would be formaldehyde detection at sub-ppm concentrations in air, using UV absorption spectroscopy in the 300–360 nm band, while providing spectral selectivity against the main interfering gases, notably NO_2_ and O_3_. Although SiN*_x_* thin films are conventionally used only for visible and near-infrared optical wavelengths (above 450 nm) because of high absorbance at lower wavelengths, this work shows that nitride-rich SiN*_x_* is suitable for near-UV wavelengths. The interplay between spectral absorbance, transmittance and reflectance in a FPI is presented in a comparative study between one FPI design using stoichiometric material (Si_3_N_4_) and two designs based on N-rich compositions, SiN_1.39_ and SiN_1.49_. Spectral measurements confirm that if the design accounts for phase penetration depth, sufficient performance can be achieved with the SiN_1.49_-based FPI design for gas absorption spectroscopy in near-UV, with peak transmission at 330 nm of 64%, a free spectral range (FSR) of 20 nm and a full-width half-magnitude spectral resolution (FWHM) of 2 nm.

## 1. Introduction

Formaldehyde (CH_2_O) is a volatile organic compound (VOC) and is known as a major cause of sick building syndrome (SBS) [1,2]; more significantly, it is a cause of inflammation of lung epithelial cells and a potential human carcinogen [3]. The World Health Organization (WHO) has recognized formaldehyde as a health hazard and has set the permissible exposure limit (PEL) at 2 ppm for short-term (15 min) exposure [4]. The US National Institute for Occupational Safety and Health (NIOSH) focuses on long-term exposure with an emphasis on the effect of accumulated exposure at the workplace and, for instance, has set the maximum long-term PEL for formaldehyde at 0.75 ppm for an 8-hr exposure [5].

Formaldehyde is one of the components that are generated in combustion, but also results from wood processing [6,7]. Drying of wood results in formaldehyde emission of up to 350 μg per m^3^ of wood (from spruce or chestnut when dried at 140 °C [8]). Moreover, emission continues for years after newly placed timber panels have become part of the interior of a home (up to 70–80 μg·m^−2^·hr^−1^ for spruce and beech) [8,9]. The possible indoor exposure levels during a vacation in a recently built all-timber cabin can be significant. Consider a simplified analysis of a family cabin with a floor area of 8 × 6 m^2^ and a roof at 2.5 m height. Assume the cabin floorplan results in an inside volume of 120 m^3^ and a total inside area of wood surface of 300 m^2^. Using a realistic value for emission from timber of 50 μg·m^−2^·hr^−1^ results in a total hourly formaldehyde generation of 15 mg. The accumulated formaldehyde concentration after an 8-h sleep amounts to 8 × 15,000/120 = 1000 μg/m^3^ = 33.3 × 10^−6^ moles/m^3^ (note that the molecular weight of formaldehyde (CH_2_O) is 30). Consequently, in the case of no ventilation, the formaldehyde exposure at wake-up is equal to 33.3 × 22.4 × 10^−3^ = 0.75 ppm (note that at standard temperature and pressure (STP) conditions, one mole of an ideal gas has a volume of 22.4 × 10^−3^ m^3^). Arguably, in the case of no ventilation, the formaldehyde exposure at wake-up could be close to the long-term PEL. In terms of the acute impact of the detection of a particular gas on health and safety, formaldehyde sensors can be classified alongside CO_2_ sensors [10,11].

A selective gas detection limit better than 1 ppb can be achieved using professional equipment for multi-component gas composition measurement. However, traditional instrumentation for multi-component gas composition measurement is bulky, with operation based on established laboratory equipment, such as gas chromatography, differential optical absorption spectroscopy (DOAS) [12,13] and laser induced fluorescence [14]. Miniaturized versions of traditional instruments, such as the gas chromatograph, have been fabricated and a detection limit at the ppb level was demonstrated [15].

Gas optical absorption spectroscopy generally makes use of an absorption line in the mid-IR wavelength. The advantages are non-contact gas sensing and the fact that a sampler is not required. In principle, the gas detectivity can be increased by increasing the path length of the light beam traveling through the gas, and high detectivity has been demonstrated [16]. Miniaturized systems are commercially available for the selective detection of CO_2_ (with characteristic absorption features in the 4.5–4.6 μm band), CO (4.5–4.6 μm band) and N_2_O (4.5–4.6 μm band) [17,18]. A low-cost implementation, the non-dispersive Infrared NDIR, is based on a heated membrane for use as a thermal emitter; in combination with a thermal detector and an absorption path length of a few cm, it is sufficient for application in air quality monitoring. However, these gases provide no usable absorption spectra in the visible/near-UV ranges. This constraint does not generally apply to a VOC such as formaldehyde, which has characteristic spectral absorption features in both the mid-IR (at 3.5 μm) and near-UV (in the 300–360 nm band) wavelengths [19,20].

Although the emphasis in optical detection of formaldehyde has traditionally also been on the use of mid-infrared spectral lines, there is a growing interest in joining the general trend towards using near-UV gas absorption spectra for the selective detection of various components of a gas [20]. Absorption spectroscopy in mid-IR is based on measuring the energy levels (wave numbers) of the various atomic vibration modes, while in UV the energy levels of the different transitions of electron states are measured. One of the consequences is the less pronounced gas spectral absorption features in UV as compared to the absorption lines in mid-IR, and the magnitude of the absorption coefficient is also lower. The advantage of measurement in near-UV is the limited overlap between the spectra of gases of interest, as compared to the relatively crowded mid-IR. Moreover, UV absorption spectroscopy in ambient air is not affected by water absorption bands, and the lower limit of the wavelength range is at about 220 nm due to oxygen absorption. In mid-IR practical operation is limited to several spectral windows in between water absorption bands. An enabling factor for miniaturized systems for absorption spectroscopy in near-UV is the availability of efficient UV LEDS, which bring significant advantages relative to heated membrane thermal emitters, which are micromachined in a silicon wafer, in terms of optical output, power consumption and costs [21].

**Figure 1:**
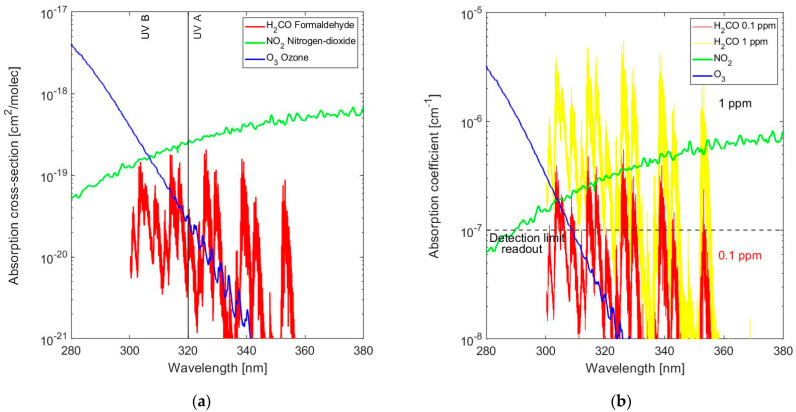
(**a**) Absorption cross-sections of formaldehyde (H_2_CO) and the two main interfering gases in near-UV: NO_2_ and O_3_; (**b**) Absorption coefficient of formaldehyde at 0.1 ppm and 1 ppm concentrations in the presence of NO_2_ and O_3_ in their concentrations in the lower atmosphere.

The optical absorption cross-section, σ, of a formaldehyde molecule in near-UV is shown in Figure 1a, in combination with that of the main interfering gases, NO_2_ and O_3_ [22,23]. The essential spectral information for the detection of formaldehyde is in the 300–360 nm band. However, this absorption spectrum overlaps with those of O_3_ and NO_2_, which are present in the lower atmosphere partly due to our collective legacy of past emissions [24]. A significant part of the concentrations of NO_2_ and NO originates from NO_x_ emissions and is associated with the generation of ozone [25]. The molecular absorption cross-section of NO in UV is in the 185–225 nm band and thus outside the part of the spectrum that is relevant to the detection of formaldehyde. The concentration of NO_2_ in air depends on amount of traffic and time of the day, but a typical value at sea level is about *Conc*(NO_2_) = 0.045 ppm [26]. Similarly, the concentration of ozone at sea level is about *Conc*(O_3_) = 0.025 ppm [27]. Combining these concentrations with the respective molecular cross sections results in the spectral absorption coefficients as shown in Figure 1b (note that: *Conc* [molecules/cm^3^] = *Conc* [ppm] × 10^−6^ × N_STP_ = *Conc* [ppm] × 2.687·10^13^ and α [cm^−1^] = σ [cm^2^/molecule] × *Conc* [molecules/cm^3^]).

The absorption coefficient for formaldehyde, α_H2CO_, at several concentrations is also included, which provides a basis for the assessment of the detection limit that is constrained by selectivity. Although the spectra of NO_2_, O_3_ and H_2_CO are overlapping, differences in the detail of the spectral information can be used to resolve the three components using data processing of the full spectral information, which is the main incentive for using high-resolution absorption spectroscopy. A concentration *Conc*(H_2CO_) = 0.1 ppm is at the threshold of being detectable and requires such optical data processing for selective detection. The objective of this work is to present N-rich SiN_x_ as a suitable material in a system realized on sapphire for achieving such a level of gas detectivity; it does not include the fabrication and testing of the actual optical gas sensor. The gas detectivity requirement is imposed by the application, and it can be concluded that UV absorption spectroscopy can be used. However, the instrument ultimately measures the change in light intensity at the optical detector due to optical absorption in a column of gas, which is set by the capability for reproducibly measuring the minimum (variation in) absorbance *A* = α*L* (with α the spectral absorption coefficient and *L* the length of the absorption path) and determines the gas detection limit depending on instrument specifications.

**Figure 2:**
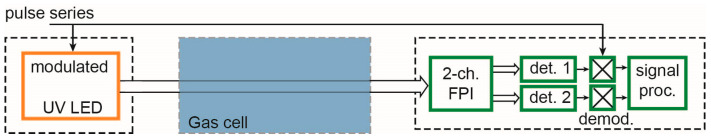
Setup of the dual-channel configuration that can be used for gas absorption spectroscopy.

Although a full instrument design is not the goal of this investigation, it is worth noting that using established techniques, such as a chopped/AC-operated light source, a DOAS set-up and coherent detection at the detector as shown in Figure 2, the gas detection limit can be improved to the point that it is limited primarily by the equivalent detection limit of the optical system, A_det,eq_, most notably by the short-term light source instability and detector noise. Typical values are from *A*_det,eq_~10^−6^ in high-end professional instrumentation to *A*_det,eq_~10^−5^ in a commercially viable consumer product. A dedicated CMOS circuit with a detector is designed for implementation in a commercially viable consumer product, and the optical detection limit is unlikely to be much better than *A*_det,eq_~10^−5^. Consequently, for the measurement of spectral absorbance with a detection limit at α_det_ = 10^−7^ cm^−1^, as shown in Figure 1b, an absorption path length *L*_min_ = *A*_det,eq_/α_det_ = 100 cm is required.

The absorption path length is one of the aspects that determines the system implementation. This path length can be realistically implemented in a system for operation in a home, but is not compatible with the dimensions of a lithographically microfabricated chip. Another system aspect that would be difficult to combine with on-chip integration is a UV LED, which usually comes as a commercial-off-the-shelf (COTS) component. Therefore, in this work a hybrid implementation is envisioned: FPIs are fabricated on sapphire wafers through PECVD of layers of SiN*_x_* and SiO_2_, maintaining the low-cost advantage in high-volume batch production, while using equipment that is available in a commercial microfabrication foundry.

## 2. Materials and Methods

### 2.1. Using Silicon Nitride as an Optical Material in Near-UV

The FPI is composed of a relatively thick optical resonator layer between two distributed Bragg reflectors (DBRs) acting as mirrors. DBRs are based on a stack of alternating dielectric layers of two different materials, with the first referred to as the high-index dielectric material (*n*_H_) and the second as the material of low index (*n*_L_). The spectral selectivity of the DBR depends on the ratio between *n*_H_ and *n*_L_, which should be maximized, and the number of layer pairs [26]. SiO_2_ is very suitable as a low-index material (*n*_L_ = 1.46–1.51) and it offers very low optical loss over most of the near-UV, visible and near-IR spectral ranges.

In a microfabrication facility, a variety of options are available for the deposition of SiO_2_, as it is widely used in electronics and MEMS. One option that is attractive for this work is plasma-enhanced chemical vapor deposition (PECVD), which is suitable for both deposition rate and film quality, with the additional benefit that it has a low thermal budget (thus, does not cause an appreciable change in the performance of active components already integrated in the substrate), which makes it attractive for back-end processes on a variety of substrates. Because of its slow deposition rate, sputtering is not a candidate if the entire FPI, including the resonator later, must be deposited in a single tool.

The selection of a microfabrication-compatible high-index DBR material presents a greater challenge. TiO_2_ is often used, because of its relatively high index *n*_H_~2.8 and low loss in the visible spectral range, which makes it a very suitable material for use in the visible wavelength; however its *k* > 0.5 for λ < 400 nm makes it generally not acceptable for use in near-UV [26]. In the 250–400 nm band, HfO_2_ is usually considered a very suitable candidate for *n*_H_ [27,28,29], and the HfO_2_/SiO_2_ combination has actually been used for realization of a microspectrometer based on a linearly variable optical filter (LVOF) [30]. Ta_2_O_5_ is in principle also a candidate [31]. In all these cases SiO_2_ remains the material of choice for *n*_L_. Combinations of fluorides, such as LaF_3_ for *n*_H_ and MgF_2_ for *n*_L_, can in principle also be considered, despite their limited CMOS fabrication compatibility, but their achievable optical index contrast with SiO_2_ is small [32,33,34].

Because of the attractiveness of PECVD as a practical, foundry-compatible microfabrication tool for the entire FPI, it is important to consider a high-index material that can be easily deposited by this tool. In this work, we focus on PECVD nitride-rich SiN*_x_* for *n*_H_ [35,36]. The choice of N-rich SiN*_x_* is not obvious. Much has been published on Si-rich SiN*_x_*, which is a low-stress material that has excellent properties for optical design in near-IR and is and has been used for waveguides operated at 1550 nm [37]. However, its optical loss for wavelengths shorter than 450 nm makes the Si-rich material unsuitable for optical design in near-UV. The near-UV application presents an additional challenge: unlike the case for the IR range, where Si-rich SiN*_x_* can be adjusted to maximize *n* by minimizing *x* without concern for optical loss, in near-UV design the adjustment of N-rich SiN*_x_* requires a carefully tuned compromise for a composition that stays above the necessary threshold of the optical index to serve as a high-index material, while staying below the threshold of the extinction coefficient for unacceptable optical loss.

**Figure 3:**
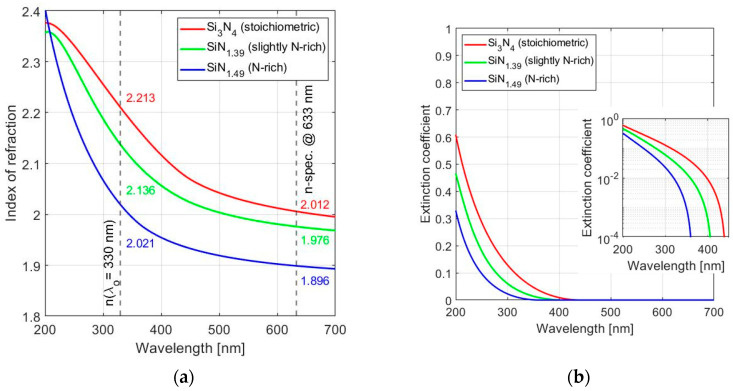
(**a**) Index of refraction, *n*, and (**b**) extinction coefficient, *k*, for PECVD Si_3_N_4_, SiN_1.39_ and SiN_1.49_ considered here, in the spectral range 200–450 nm. These curves are extracted from ellipsometry data (Woollam M-2000) using the Tauc–Lorentz model with mean squared error (MSE) of 3.10, 1.93 and 3.83 respectively.

In our earlier work we found N-rich SiN*_x_* with *x*~1.49 to be an optimum composition when also considering the repeatability of the deposition rate [36]. This material provides good optical performance in the near UV (300–400 nm), with *n*~2.02 and *k* < 10^−2^ at the design wavelength λ_o_ = 330 nm. The spectral dependence of *n* and *k* for the different compositions of SiN_x_ used in this work is shown in Figure 3.

### 2.2. Fabry–Perot Interferometer Design

In its most generic representation, an FPI is composed of a resonator in between two identical reflectors, each with a transmission coefficient *t* (transmittance *T* = *t*·t*) and reflection coefficient *r* (reflectance *R* = *r*·r*). The overall transmittance through the FPI, *T*_FPI_, is the transmitted optical irradiance *I*_t_ relative to the incident intensity *I*_i_, and can be derived by collecting all transmitted wave components after (multiple) reflection at the two mirrors, and results at normal light incidence in [38]:

(1) TFPI=ItIi=T2R2−2Rcos⁡δ+1=T21−R2×11+4R1−R2sin2⁡δ2=T21−R2×11+Fsin2⁡δ2

with δ the round-trip phase delay, δ = 4π*n*_res_(λ) t_res_/λ for a light beam after traveling from the mirror of entrance into the resonator layer, being reflected at the second mirror and returning to the mirror of incidence, and *F* = 4*R*/(1 − *R*)^2^ the coefficient of finesse. The peak transmission is at sin^2^(δ/2) = 0, which is equivalent to δ_max,m_/2 = 2*m* × π/2 → δ_max,m_ = *m* × 2π with *m* the order of the resonance. Consequently:

(2) $$\lambda_{max,m}=\frac{4\pi n_{res}t_{res}}{\delta_{max,m}}=\frac{2n_{res}t_{res}}{m},$$

Designing for operation at a high-order mode is advantageous, because the selectivity of the filter is defined by the spectral resolution (the full-width half magnitude bandwidth at order *m*, *FWHM*_m_), which can be expressed as:

(3) $${FWHM}_{m}\sim \lambda_{max,m}\times \frac{2}{m\times \pi \sqrt{F}},$$

Therefore, *FWHM*_m_ scales down with order *m*. The minimum usable mode results from the specifications and a reasonably achievable value for *F* when considering the fabrication technology. The near-UV spectral absorption characteristics of formaldehyde, as presented in Figure 1, result in a mid-band resonance wavelength at about λ_max,m_ = 330 nm and *FWHM*_m_~1–2 nm. For *F*_max_~200 (*R*~0.86) the minimum operating order required for meeting the specification on spectral resolution *m*_min_ = (λ_max,m_/*FWHM*_m_)_spec_ × (2/π√*F*_max_) = (330/1) × (0.045)~15. However, FPI optical design should also meet the required spectral spacing between peak transmissions, the free spectral range at order *m FSR*_m_:

(4) $${FSR}_{m}=\lambda_{max,m}-\lambda_{max,m+1}=2n_{res}t_{res}\left(\frac{1}{m}-\frac{1}{m+1}\right)=\frac{\lambda_{max,m}}{m+1},$$

As a consequence, *m* = 15 results in *FSR*_15_ = 330/16 = 20.6 nm, which is smaller than the full band containing relevant information about formaldehyde, but at this stage can be considered adequate for its selective detection by capturing the gas-specific spectral signature. It should be noted that the ratio *FSR*_m_/*FWHM*_m_ = (π/2)(*m* + 1)√*F*_max_/*m*, which for *m*»1 primarily depends on *F*. Obviously, more demanding specifications can be met in a design or technology that provides higher mirror reflectance and thus higher *F*.

**Figure 4:**
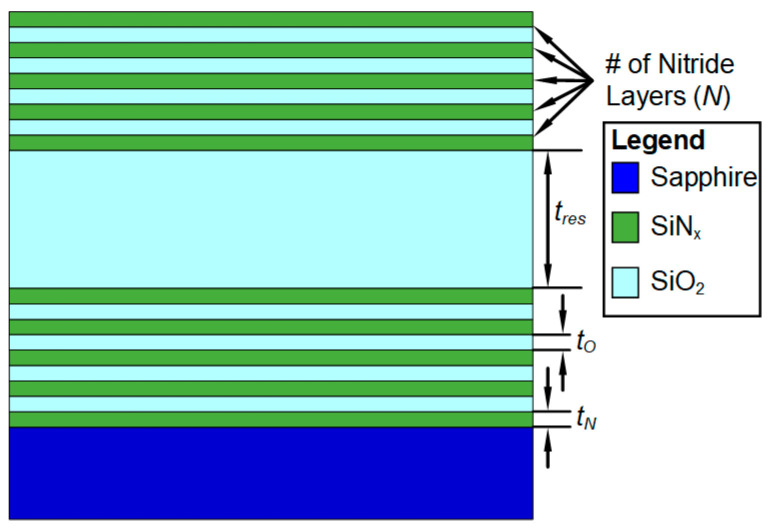
Structure of a Fabry–Perot interferometer using 9-layer distributed Bragg reflectors with SiN_x_ (*t*_H_ = *t*_N_) as the high-index material and SiO_2_ as the low-index material (*t*_L_ = *t*_O_).

In the most-conventional configuration, a distributed Bragg reflector (DBR) is used as the implementation of each of the mirrors, which results in the FPI structure shown in Figure 4. The FPI design presented is composed of a 19-layer structure, with the 9-layer lower DBR directly on top of the sapphire substrate and the same 9-layer DBR design forming the top of the structure and facing the medium of incidence (air) with a thick resonator layer in between. A DBR is generally composed of a stack of layers of alternatingly high-index material of thickness *t*_H_ and low-index material of thickness *t*_L_, while the resonator, with thickness *t*_res_, is of the low-index material. In this work SiN_x_ was used as the high-index material (*t*_H_ = *t*_N_) and SiO_2_ as the low-index material (*t*_L_ = *t*_O_) and the layer thicknesses are referred to in the following as *t*_N_ and *t*_O_.

The input admittance of the overall optical system composed of the stack of thin films on a substrate can be defined as *y*_sys_ = *n*_sys_ × *y*_fs_ = C/B, with *y*_fs_ = (ε_o_/μ_o_)^1/2^ the admittance of air (free space), parameter B the ratio between the electric field component at the incident side of the thin film (at the interface between the medium of incidence and the film) and that at the exit side of the thin film (at the interface between the film and the substrate), and C as the ratio between the magnetic field component at the incident side of the thin film and that at the exit side of the thin film. The notation in terms of admittances can be used to derive the generalized expression for the coefficient of reflectance of the overall system, *r*_sys_ [38]:

(5) $$r_{sys}=\frac{y_{fs}-C/B}{y_{fs}+C/B}=\frac{y_{fs}-y_{sys}}{y_{fs}+y_{sys}}.$$

For a system comprising a single film, B and C can be calculated by considering the continuity requirements of the tangential (in-plane) components of the incident and reflected fields at the optical interface of incidence (between air and the film). For a single film with admittance *y*_2_, on a substrate with admittance *y*_sub_ [38]:

(6) $$\left[\begin{array}{c} B\\ C \end{array}\right]=\left[M\right]\cdot \left[\begin{array}{c} 1\\ y_{sub} \end{array}\right]=\left[\begin{array}{cc} \cos\left(\delta /2\right) & j\frac{\sin\left(\delta /2\right)}{y_{2}}\\ jy_{2}\sin\left(\delta /2\right) & \cos\left(\delta /2\right) \end{array}\right]\cdot \left[\begin{array}{c} 1\\ y_{sub} \end{array}\right],$$

with *M* the characteristic matrix of the single-layer system and (δ/2) = 2π*n*_2_(λ)*t*_2_/λ the one-way phase shift upon traversal of the film (half the round-trip phase delay δ for light traveling through the layer). In the FPI design only two materials were used: SiO_2_ with *n*_L_ = *n*_O_ and admittance *y*_2L_, and SiN*_x_* with *n*_H_ = *n*_N_ and admittance *y*_2H_. Their respective characteristic matrices are:

(7) $$\left[M_{L}\right]=\left[\begin{array}{cc} \cos\left(\delta /2\right) & j\frac{\sin\left(\delta /2\right)}{y_{2L}}\\ jy_{2L}\sin\left(\delta /2\right) & \cos\left(\delta /2\right) \end{array}\right];\left[M_{H}\right]=\left[\begin{array}{cc} \cos\left(\delta /2\right) & j\frac{\sin\left(\delta /2\right)}{y_{2H}}\\ jy_{2H}\sin\left(\delta /2\right) & \cos\left(\delta /2\right) \end{array}\right].$$

For an effective DBR, each layer in the stack should be at the resonance for the design wavelength: δ/2 = π/2. Therefore, the layer thickness is tuned *n*_H_(λ_o_)*t*_N_ = *n*_L_(λ_o_)*t*_O_ = λ_o_/4, which is referred to as the quarter-wavelength optical thickness (QWOT), *t*_QWOT_. In conventional optical filter design, materials are usually selected for which optical losses in the targeted spectral operating range can be disregarded (*k* = 0 and δ depends on n* = *n*) and the diagonal elements in *M*_L_ and *M*_H_ are zero. This results in a significant simplification and enables the derivation of analytical expressions for the peak reflectance.

The loss in near-UV is explicitly not disregarded and is accounted for in the extinction coefficient, *k*, of the complex index of refraction, n* = *n* − j*k*. Therefore, the admittance of a layer is also a complex number. The diagonal matrix elements are non-zero complex numbers and calculation of the spectral reflectance of the 9-layer DBR and the transmittance of the 19-layer FPI requires numerical matrix multiplication:

(8) $$\begin{array}{c} \left[\begin{array}{c} B\\ C \end{array}\right]=\left[M_{DBR}\right]\cdot \left[M_{res}\right]\cdot \left[M_{DBR}\right]\cdot \left[\begin{array}{c} 1\\ y_{sub} \end{array}\right],with\\ \left[M_{DBR}\right]=\left[M_{H}\right]\cdot \left[M_{L}\right]\cdot \left[M_{H}\right]\cdot \left[M_{L}\right]\cdot \left[M_{H}\right]\cdot \left[M_{L}\right]\cdot \left[M_{H}\right]\cdot \left[M_{L}\right]\cdot \left[M_{H}\right] \end{array}$$

with *M*_H_ and *M*_L_ the characteristic matrix of a QWOT layer of the high-index material and low-index material, respectively, and *M*_res_ the characteristic matrix of the resonator layer with its optical thickness tuned in principle to the 15th-order resonance: *n*_L_(λ_o_)*t*_res_ = *m*λ_max,m_/2 = 7.5λ_max,m_. The layer thickness for a DBR with lossy layers is set to *t*_QWOT_, which provides resonance at λ_o_, but the actual layers do in addition result in a loss component. From Equation (8) the reflection coefficient *r*_sys_ = *r*_FPI_ can be calculated, from which the expressions for spectral reflectance *R*_FPI_(λ), transmittance *T*_FPI_(λ), and absorbance *A*_FPI_(λ) of the FPI result. These are presented in the literature and are not reproduced here [38] (p. 56).

The approach used for simulations of the spectral response of the various options for a FPI is based on the direct implementation in Matlab of a routine for solving Equations (7) and (8) over a user-defined wavelength range and with a database of the extracted indexes of refraction and extinction coefficients of the different SiN*_x_* compositions used (as shown in Figure 3 and listed in Table 1). Literature data on sapphire was used: *n*_Al2O3_ = 1.81 ± 0.04 in the range 250 nm to 800 nm, while the extinction coefficient *k*_Al2O3_ in that range can be disregarded [39]. This customized software allows the simultaneous presentation of spectral reflectance *R*_FPI_(λ), transmittance *T*_FPI_(λ), and absorbance *A*_FPI_(λ), which provides essential insight into their interplay, especially between transmittance and absorbance, at different material compositions; this information cannot be easily extracted when using commercially available software packages for optical design.

A complication in specifying the actual operating mode of the resonator is the fact that the mirroring plane of a DBR is not the inner front surface of the DBR, as is often assumed in FPI design, but instead a position deeper in the stack due to phase penetration [40,41,42]. Consequently, the FPI is actually operated at a higher mode than anticipated and incorrect conclusions are drawn from device performance when verifying the measured properties with Equations (3) and (4). Research on the position of the mirror plane within the DBR in terms of effective phase penetration depth, *L*_pp_, demonstrates a dependence on the refraction index contrast of the materials used for the QWOT layers, the number of layer pairs in the DBR, and the design wavelength. From literature and the materials and dimensions used in our design, the equivalent mirror position is estimated at a distance 0.6 × λ_o_ < *L*_pp_ < 0.8 × λ_o_ from the interface between the resonator layer and the first inner *n*_H_ layer of the DBR. In the designs presented, we assume *L*_pp_ = 0.75 × λ_o_ = 3*t*_QWOT_ and we attempt to derive actual data from our experimental results.

**Figure 5:**
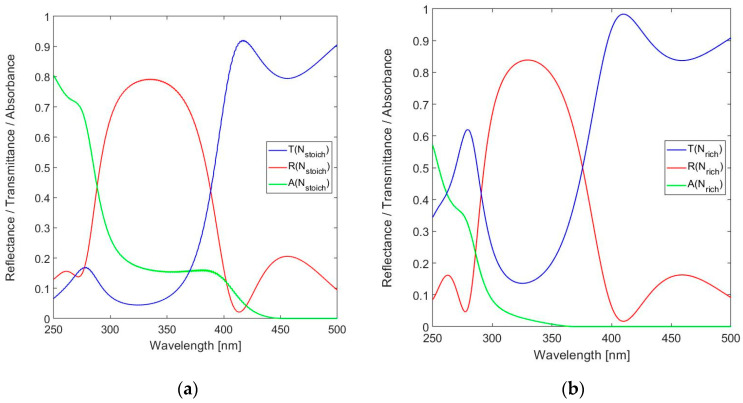
Calculated spectral reflectance, transmittance and absorbance for the 9-layer DBR in the near-UV to short-wavelength visible spectral range (250–500 nm) for: (**a**) Stoichiometric SiN*_x_* (Si_3_N_4_) and (**b**) N-rich SiN*_x_* with *x* = 1.49.

As a consequence, the nominal optical resonator thickness *n*_L_*t*_res_ should be reduced from 15 × λ_o_/2 = 2475 nm to 15 × λ_o_/2 − 2 × 0.75 × λ_o_ = 1980 nm for operation at *m* = 15 at λ_o_ = 330 nm. The physical thicknesses of the layers used in the final FPI designs are listed in Table 1. The performance of the FPI strongly depends on the DBR design, which should provide a sufficiently high peak reflectance at the design wavelength while the bandwidth of the reflectance band should be sufficiently wide to capture the band that contains the specific information on formaldehyde. As indicated, the starting point is a 9-layer DBR. Figure 5a shows the spectral reflectance, transmittance and absorbance for a stack of Si_3_N_4_ based *n*_H_ QWOT layers alternating with SiO_2_ QWOT layers for *n*_L_. Similarly, Figure 5b shows these spectral responses for N-rich SiN*_x_*-based QWOT layers for *n*_H_. 

The half-peak reflection band Δλ(SiN_1.49_) = 380.3 − 91.0 = 89.3 nm is sufficiently wide for spectrally covering the three resonance modes of the FPI. In a final design, the DBR reflectance band should preferably be designed for narrowly containing one resonance band to minimize out-of-band detection, but in this work a wider band is preferred for analyzing the effect of lossy layers using the transmittance peaks at several modes. The peak reflectance of the N-rich design is slightly higher compared to the stoichiometric material (*R*(330 nm) = 0.839 instead of 0.793), but the differences in transmittance and absorbance are more significant. While the absorbance at the design wavelength *A*(330)~2% for SiN_1.49_, *A*(330 nm) > 15% for Si_3_N_4_. The lower absorbance, combined with the higher reflectance of the N-rich material, is clearly an advantage and results in a DBR of higher performance. However, the peak reflectance of 0.84 is not impressive.

**Figure 6:**
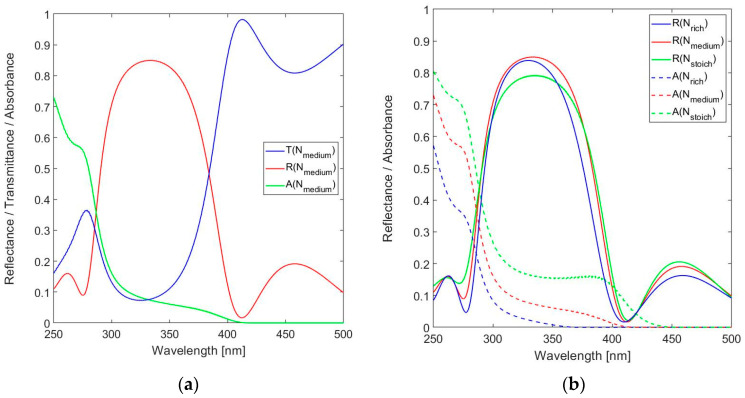
(**a**) Calculated spectral reflectance, transmittance and absorbance for 9-layer DBR in the near-UV to short-wavelength visible spectral range (250–500 nm) for SiN*_x_* with *x* = 1.39 and (**b**) spectral reflectance and absorbance for different SiN*_x_* compositions.

The compromise between high reflectance and low absorbance is very subtle, which becomes apparent when also considering SiN_x_ of intermediate composition (*x* = 1.39). The spectral reflectance, transmittance and absorbance of SiN_1.39_ is shown in Figure 6a. The half-peak reflection band Δλ(SiN_1.39_) = 387.1 − 288.3 = 98.8 nm and, as expected, is wider compared to that of SiN_1.49_. However, peak reflectance is also slightly higher (*R*(330 nm) = 0.848 for SiN_1.39_ versus 0.839 for SiN_1.49_), which is not obvious. As shown in Figure 6b, the in-band absorbance for SiN_1.39_ decreases to about the same value as the transmittance (8%), which implies that the advantage of its higher refractive index starts to outweigh the adverse effect of absorbance on the DBR reflectance for λ > λ_o_. The compromise between refraction index contrast and layer absorbance is skewed toward favoring the former in the second-order reflectance peak at 450 nm, because absorbance does not play a significant role in that spectral range. Consequently, the overall specifications of a 9-layer DBR reflector in a SiN_1.39_-based design are slightly superior to those of a SiN_1.49_-based design. However, the next simulations show that the performance metrics of the 19-layer FPI design are not well served by DBRs based on SiN_1.39_ and the N-rich composition, SiN_1.49_, should be used for *n*_H_.

**Figure 7:**
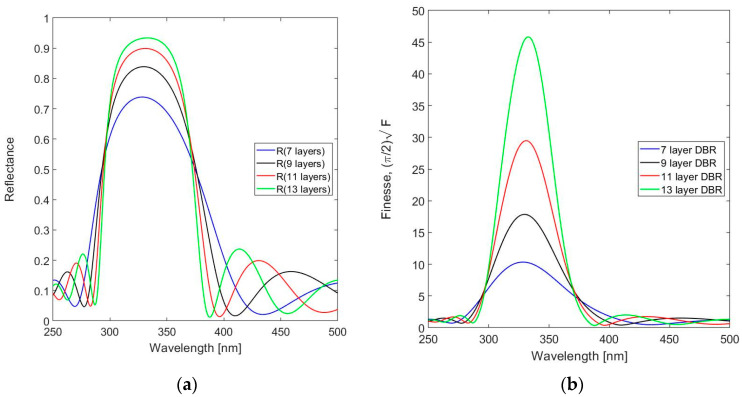
Effect of the number of *n*_H_ layers of SiN_1.49_ in the stacked QWOT layer stack on (**a**) DBR spectral reflectance and (**b**) Finesse.

The optical performance of the DBR strongly depends on the number of stacked QWOT layers, as is confirmed in Figure 7. 

**Figure 8:**
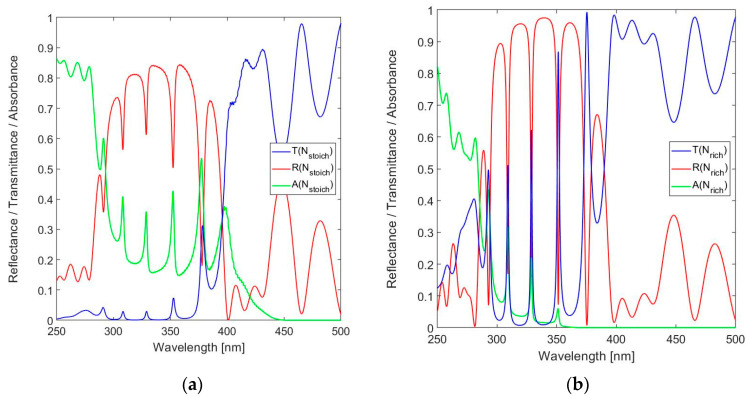
Calculated spectral reflectance, transmittance and absorbance for 19-layer FPI in the near-UV to short-wavelength visible spectral range (250–500 nm) for: (**a**) Stoichiometric SiN*_x_* (Si_3_N_4_) and (**b**) N-rich SiN*_x_* with *x* = 1.49.

As expected, the peak reflectance and the associated Finesse increase with the number of layers in the DBR. The 9-layer DBR design is taken as the basis for simulations of the FPI spectral response. The following simulations all target a design with a resonator thickness *t*_res_ = 1320 nm and *m* = 15. The spectral reflectance, transmittance and absorbance of the 19-layer FPI with Si_3_N_4_ used for the *n*_H_ layers are shown in Figure 8a and those for SiN_1.49_ in Figure 8b.

Figure 8 confirms the three modes of resonance within the DBR bandwidth and the superior spectral performance of the N-rich implementation of the *n*_H_ layers. The peak transmission improves from *T*_FPI_(328.6 nm) = 0.028 for Si_3_N_4_ to *T*_FPI_(328.8 nm) = 0.623 for SiN_1.49_. The peak transmission for Si_3_N_4_ is obviously of little practical use, because of the significant absorbance *A*_FPI_(328.6 nm) = 0.359, while *A*_FPI_(328.8 nm) = 0.222 for SiN_1.49_. The SiN_1.49_-based FPI features an FWHM(328.8 nm) = 1.4 nm. The peak transmission *T*_FPI_(351.2 nm) = 0.860 is even higher compared to that at the primary design wavelength, which is due to the reduction in absorbance to *A*_FPI_(351.2 nm) = 0.059. The envelope of the spectral reflectance is in good agreement with the DBR spectral reflectance, as shown in Figure 5. However, the FWHMs of the two additional transmission peaks within the BDR bandwidth are larger, FWHM(309.0 nm) = 1.7 nm and FWHM(351.2 nm) = 2.2 nm, which is the result of fall-off of the spectral reflectance of the DBR at these wavelengths. 

**Figure 9:**
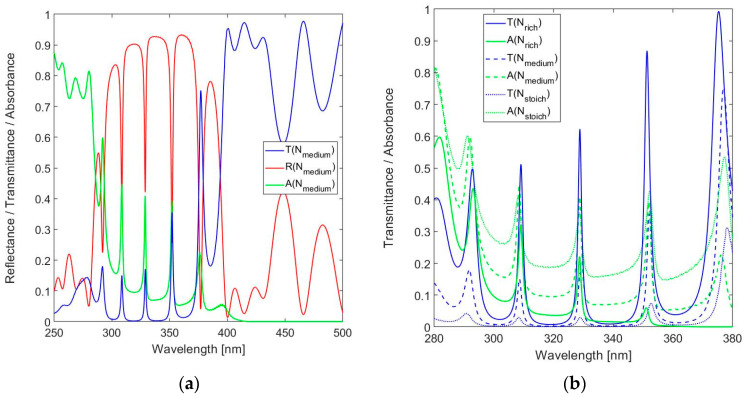
(**a**) Calculated spectral reflectance, transmittance and absorbance for 19-layer FPI in the near-UV to short-wavelength visible spectral range (250–500 nm) for SiN*_x_* with *x*= 1.39 and (**b**) spectral transmittance and absorbance for the different SiN*_x_* compositions.

For validation, the spectral response of the FPI with SiN_1.39_ used for the *n*_H_ layers was also computed and the result is shown in Figure 9a. The spectral performance of the SiN_1.39_-based FPI is, as expected, clearly in between that of the Si_3_N_4_-based implementation and the one based on SiN_1.49_. The peak transmission *T*_FPI_(328.8 nm) = 0.170. Although the FWHM(328.8 nm) = 1.5 nm is very close to that of SiN_1.49_, the in-band absorbance *A*(328.8 nm) = 0.409 exceeds even that of the Si_3_N_4_-based FPI. These are remarkable aspects and result from the higher reflectance of the SiN_1.39_-based DBR, which outweighs the effect of the higher loss.

The curves are combined in Figure 9b for enabling a comparison and confirm that, despite the higher *R*_DBR_(328.8 nm) of the SiN_1.39_-based DBR compared to its SiN_1.39_-based counterpart, this advantage does not outweigh the adverse effect of loss in the layers in the respective FPI realizations.

**Figure 10:**
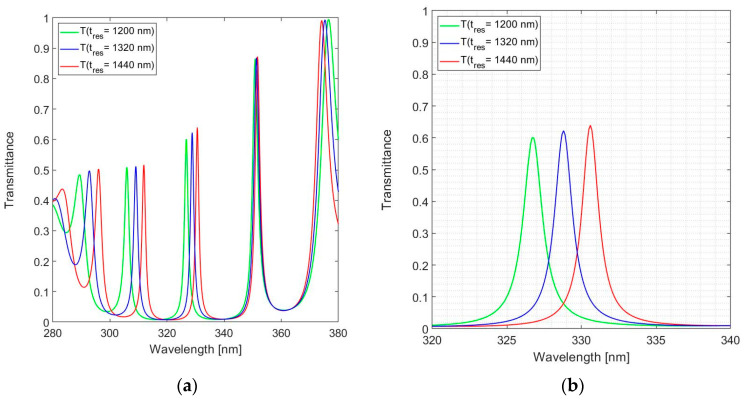
Calculated spectral transmittance for the 19-layer FPI with SiN_1.49_ used for *n*_H_: (**a**) Full near-UV spectral range and (**b**) zoom in around the design wavelength.

The spectral responses shown in Figure 8 and Figure 9 refer to an FPI operated at *m* = 15 with a physical resonator thickness of 1320 nm. The transmission curves for the three operating modes considered here are shown in Figure 10. 

The magnitude of the peak transmittance varies with the reflectance of the DBR. The transmittance in the 330 nm band ranges from *T*(326.8) = 0.600 and *T*(328.8) = 0.623 to *T*(330.6) = 0.639, with FWHM(326.8) = 1.6 nm, FWHM(328.8) = 1.4 nm and FWHM(330.6) = 1.3 nm. The improved resolution of the FPI with a thicker resonator is consistent with operation at a higher mode number. This conclusion is also confirmed by the differences in spectral spacing between the transmittance peaks (their respective free-spectral ranges), for instance, from 18.8 nm between the 330 nm band and the 309 nm band for a 1440 nm resonator, to 20.8 nm for a 1200 nm resonator. Equation (3) can be applied to confirm that the differences are due to the different modes of operation of these resonators in the various bands, leading to the gradual encroachment of the transmittance curves in the 350 nm band and ultimately in the reversal of the relative spectral positions in the 375 nm band.

These simulations all target a design with a resonator thickness *t*_res_ = 1320 nm and *m* = 15. For validation purposes, designs with *t*_res_ = 1200 nm and *t*_res_ = 1440 nm were considered as limiting cases; only these two designs, as specified in Table 1, were actually fabricated and subjected to tests.

### 2.3. Fabrication and Measurement Setup

Plasma-enhanced chemical vapor deposition (PECVD), with silane and ammonia as the precursor gases, was used for fabrication. The deposition tool used was the GSI ULTRADEP 2000, which was operated at 350 °C and was configured as a single chamber with a load lock [34]. The ammonia gas flow was set to 550 sccm and three different recipes were used for setting the silane flow to the appropriate values to result in the different SiNx compositions. The silane flow was set to 84 sccm for Si_3_N_4_, 70 sccm for SiN_1.39_ and 45 sccm for SiN_1.49_. It should be noted that the total gas flow was not kept constant between the different recipes. The resulting materials were optically characterized using an ellipsometer setup, as already described and with results shown in Figure 3. The recipes for deposition were fully characterized [36]. The spectral transmittance and reflectance of the different fabricated versions of the FPI were measured.

**Figure 11:**
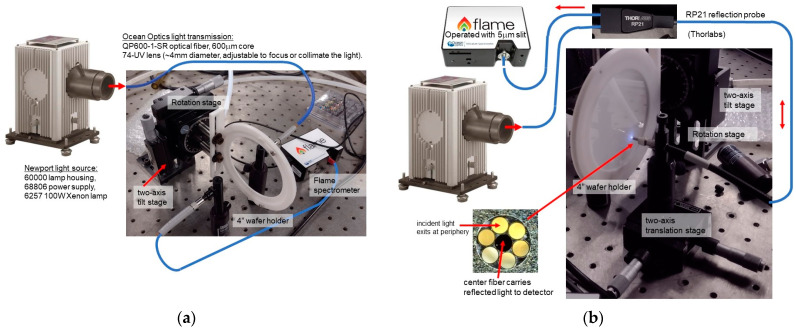
Setup used for FPI optical characterization: (**a**) Measurement of transmittance: Tilt and rotation stages were used for selecting a suitable position on the wafer and for adjusting the angle of incidence to normal incidence; (**b**) Reflectance measurement setup using a reflection probe with the reflected light guided in the inner fiber and the illuminating light in the surrounding fibers. The tilt and rotation stages were used for selecting a suitable position on the wafer and for setting the angle of incidence.

In the setup for transmission measurement, shown in Figure 11a, a fiber-based probe was used for illuminating the sample with light from a wideband light source (Newport Corporation) and for directing the transmitted light to a spectrometer (Flame, Ocean Optics). Tilt and rotation stages were used for selecting a suitable position on the wafer and for adjusting the angle of incidence for normal incidence. The lamp spectral radiation was measured separately before and after a separate spectral transmittance measurement for reducing the effect of any non-reproducibility due to the lamp, using normalization in software. Although sequential measurements do introduce uncertainty compared to direct measurement using a beam splitter, this approach is preferred for practical reasons and the uncertainty is mitigated by systematically checking the consistency of subsequent lamp spectral radiation measurements.

The transmittance through a sample was measured, which included the exitance of the sapphire–air interface at the backside of the sapphire wafer. However, the transmittance of the simulations presented refer to the radiance entering the sapphire substrate. The difference is mainly because no detector was yet integrated into the substrate. This effect was accounted for by a calibration measurement of the transmittance through a sapphire prime wafer (which basically served as the reference element). The transmittance through such a single sapphire wafer (lossless and without roughness on frontside and backside) surrounded by air is described by *T*_air,Al2O3,air_ = (1 − reflectance at the air-sapphire incidence) × (1 − exitance at the sapphire–air interface). This results in a transmittance equal to (1 − *R*_air,Al2O3_) × (1 − *R*_Al2O3,air_) = 0.841 for *n*_Al2O3_ = 1.81 and *k*_Al2O3_ = 0 (approximation based on [39]). As frontside reflectance and backside exitance can be assumed to be equal, the square root of the measured spectral transmittance through this prime sapphire prime wafer, (*T*_air,Al2O3,air_)^1/2^, can be used as a normalization factor and can be applied to the results on FPI samples.

With minor modifications the measurement setup was also used for FPI spectral reflectance measurement, as shown in Figure 11b. The most significant difference was the use of the reflection probe (Thorlabs) with a special multi-fiber probe for illuminating the sample and for transferring the reflected light. A translation stage was used for selecting a suitable location on the wafer for measurement, while a rotation stage was used to ensure normal incidence. A 20D10AL.2 UV/visible reference reflector was used for calibration (Newport Corporation). The transmittance and reflectance measurements were taken sequentially, which does in principle introduce uncertainty in the exact testing location on the wafer to be illuminated and deviations in the angle of incidence. However, this limitation had no apparent effect on our results. Therefore, these are not addressed here in more detail, but are avoidable in a more advanced setup.

## 3. Results

**Figure 12:**
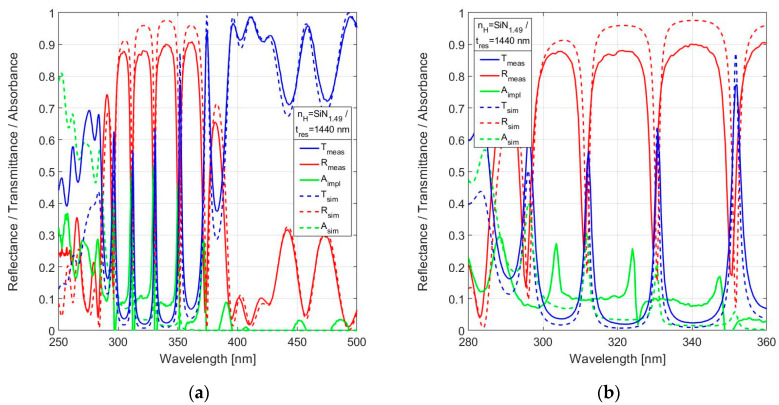
Measured spectral reflectance and transmittance with implied absorbance in comparison with simulated responses for 19-layer FPI with SiN_1.49_ used for *n*_H_ and a nominal resonator width *t*_res_ = 1440 nm in the range (**a**) 250–500 nm, (**b**) 280–360 nm.

The measured spectral reflectance and transmittance of nitride rich SiN_1.49_ with *t*_res_ = 1440 nm are shown in Figure 12 in comparison with simulation results. 

The absorbance was not measured, but is derived from the measured transmittance and reflectance by implication using *A*_impl_ = 1 − *T*_meas_ − *R*_meas_. The spectral measurements are in reasonable agreement with simulations. The in-band peak transmission in the primary design band λ_o_ = 320–330 nm is T(330.8 nm) = 63.1%, which is adequate for practical use. The spectral resolution *FWHM*(331 nm) = 1.9 nm is slightly inferior to the theoretical prediction of *FWHM* = 1.4 nm. The comparison of theory and results suggest a high accuracy in the settings of the deposition parameters, as the actual resonator thickness was very close to the nominal value.

**Figure 13:**
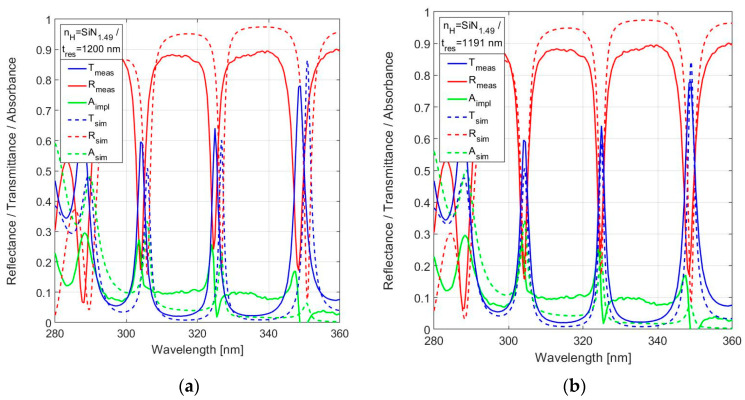
Measured spectral reflectance and transmittance with implied absorbance in the range 280–360 nm, in comparison with simulated responses, for 19-layer FPI with SiN_1.49_ used for *n*_H_ for: (**a**) the nominal resonator width *t*_res_ = 1200 nm and (**b**) the adjusted resonator width *t*_res_ = 1191 nm for best fit between measurement and simulation.

However, the results on the same nitride-rich SiN_1.49_ with resonator thickness at nominal value *t*_res_ = 1200 nm, as shown in Figure 13a, give reason for a more cautious conclusion. These measurements are also presented in Figure 13b in comparison to simulations with the resonator thickness adjusted for best fit. The results suggest an actual resonator thickness *t*_res_ = 1191 nm. The deviation of 9 nm (<1%) due to tolerances in the equipment remains fully acceptable. The in-band peak transmission of the FPI with thickness *t*_res_ = 1191 nm is T(324.9 nm) = 64.1%. The *FWHM*(324.9 nm) = 2.2 nm, which is slightly inferior to the predicted simulation value of 1.5 nm. The reduced spectral resolution (higher value of the *FWHM*) is consistent with the lower resonance mode *m* in an FPI with shorter resonator thickness.

Figure 13a reveals flattening of the peak as a source of uncertainty in the measured magnitude of the peak transmission at a resonance wavelength (for instance at λ = 304.2 nm and λ = 348.7 nm). This is the result of the step size in the spectrometer used, which basically results in an occasional pass over of the precise value of the peak wavelength. The effect is considered acceptable within the framework of this study and can be reduced in an improved measurement setup.

**Figure 14:**
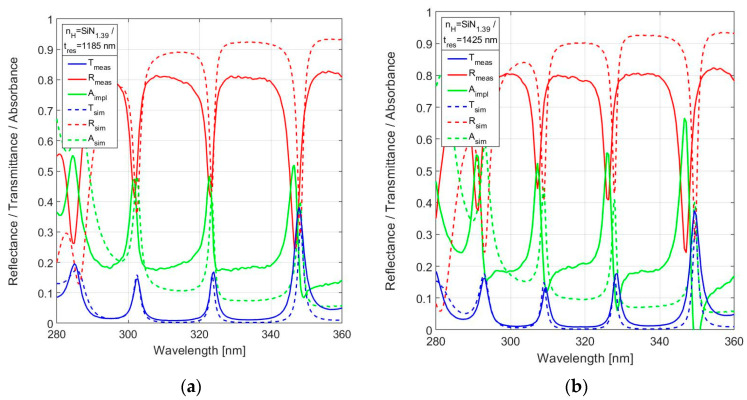
Measured spectral reflectance and transmittance with implied absorbance in the range 280–360 nm, in comparison with the simulated responses for 19-layer FPI with SiN_1.39_ used for *n*_H_ for (**a**) *t*_res_ = 1185 nm and (**b**) *t*_res_ = 1425 nm.

The measured spectral reflectance and transmittance of SiN_x_ with a medium nitride composition (SiN_1.39_) is shown for nominal resonator thickness *t*_res_ = 1200 nm and *t*_res_ = 1440 nm in Figure 14a and 14b, respectively. The results within the spectral range up to 340 nm show peak transmittance in the range 0.13–0.16, while the peak transmittance at 348 nm is about 0.38. This is a rather marginal optical performance for practical application.

**Figure 15:**
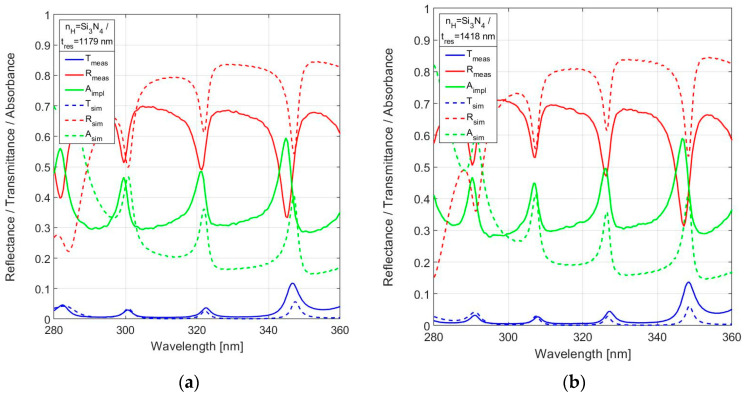
Measured spectral reflectance and transmittance with implied absorbance in the range 280–360 nm, in comparison with simulated responses, for the 19-layer FPI with Si_3_N_4_ used for *n*_H_ for (**a**) *t*_res_ = 1179 nm and (**b**) *t*_res_ = 1418 nm.

Finally, the measured spectral reflectance and transmittance of stoichiometric silicon-nitride (Si_3_N_4_) is shown for nominal resonator thickness *t*_res_ = 1200 nm and *t*_res_ = 1440 nm in Figure 15a and Figure 15b, respectively. As expected, the low transmittance (<5%) and high in-band absorbance (up to 50%) of the FPIs based on Si_3_N_4_ makes these unsuitable for a design operating at a design wavelength lower than 400 nm. The results also confirm the reduced out-of-band reflectance due to layer absorption.

## 4. Discussion

The results clearly confirm the superior performance of an FPI operating in near-UV with nitride-rich SiN_x_ used for higher-index layers in the DBRs, in comparison to Si_3_N_4_. The in-band peak transmission for λ_o_ = 320–340 nm improves from *T* < 5% in Si_3_N_4_ to *T* > 60% for SiN_1.49_, while the out-of-band peak reflectance increases from *R* < 70% in Si_3_N_4_ to *R* > 85% for SiN_1.49_. The spectral characteristics within the DBR reflectance band of the FPI designs fabricated and tested in this work are summarized in Table 2.

The measured spectral positions of the peak in-band transmission are in good agreement with simulations. It should be noted, of course, that this does not come as a surprise, as the estimated thickness of the resonator layer is adapted for best fit. From comparison between measurement and simulation, the actual thickness of the thick resonator is considered equal to the nominal value, *t*_res_ = 1440 nm (see also Figure 12), while the estimation of the thin resonator is *t*_res_ = 1191 nm (see also Figure 13). The table confirms a remaining uncertainty ε < 0.7%. Note the increased minimum transmission in between resonance peaks. This baseline increases in the *t*_res_ = 1192 nm design from the simulated *T* = 0.009 to *T* = 0.020 in the measurements and is due to parasitic transmission paths.

The measured spectral resolution is systematically smaller that the simulation results (*FWHM*_m_(meas) > *FWHM*_m_(sim), which is due to limiting aspects of the device and the measurement setup. The theory of the FPI is based on two opposing mirrors that are positioned perfectly parallel and have no surface roughness. Practical device limitations have an adverse effect on Finesse [43,44]. The significance of these imperfections is related to wavelength and thus increases with a smaller design wavelength. As a consequence, this effect is more significant in near-UV compared to microfabricated devices reported in the literature, which are typically designed for application in visible or near-IR spectra. The alternative reason for the reduced spectral resolution in measurements is the use of a spectrometer in the setup. The spectrometer is used with a slit of 5 μm width, which is the minimum value available and results in a measurement resolution of 0.95 nm (grating range 650 nm, detector array with 2048 pixels and a pixel resolution of 3 pixels [45], p. 38). The FPI presented in this work is based on an all solid-state design, rather than an airgap for use as the resonator, and we do not expect mirror parallelism or surface roughness to be the most significant limitation. The reduced spectral resolution observed is mainly due to the measurement setup and better matching with the simulations should be possible in an improved setup. Although improved consistency between simulation and experiment would of course be useful for device characterization, we would like to emphasize that the FPI is intended for use in an optical system with similar specifications as those of this measurement setup. Therefore, we tend to conclude that an FWHM of about 2 nm would be the ultimate final performance of this FPI design, which is sufficient for the intended application.

In the absence of rounding, the derived resonance mode, *m*_der_, and *FSR* can be calculated from the data in Table 2. The FSR is defined in Equation (4) as a function of the resonance wavelength (and thus of the optical width of the resonator), but also as the spectral difference between two subsequent resonant peaks. The actual values listed in Table 2 provide the information needed to verify the validity of the assumed position of the equivalent mirror within the DBR stack, which accounts for the penetration depth, by validation of resonator mode *m*. Of course, the actual value of *m* is an integer number. The spectral separation of the resonance peaks is in good agreement with theory, which confirms the correct operating order, but the derived order *m*_der_ is systematically larger than the target value. This implies that the resonance wavelength is slightly shifted to longer wavelengths, which suggests that the assumption of a mirror at 3 × *t*_QWOT_ = 0.75 × λ_o_ is a slight overestimation and a position at about 2.6 × *t*_QWOT_ = 0.65 × λ_o_ would be more appropriate. It should be noted that the actual operating mode at a given design wavelength results from device design and is not due to the characteristics of the light source used for exciting resonance. The higher-than-expected resonance mode often goes unnoticed and the discrepancy between theoretical and experimental values of the FSR is often attributed to loss mechanisms that were not taken into account in the design phase, rather than to disregarding the phase penetration depth. The results presented do support the conclusion that the effect of phase penetration at the DBRs needs to be included in an accurate FPI design.

The detrimental effects discussed can be attributed to the loss in the SiN_x_ layers. This conclusion triggers the question whether a further improvement would be possible by a further reduction of the nitride composition in SiN_x_. In principle, reduced optical loss in near-UV with a larger nitride composition should be feasible; however, there are two practical constraints to consider. The first is the deposition uniformity. An increase in nitride content beyond *x*~1.5 would, in principle, enable a push of the lower wavelength limit of the design window deeper into the UV. However, this ambition would be ultimately constrained by the manufacturing repeatability of the deposition rate and the resulting uncertainties in the composition of the deposited material. This limitation depends on the specifics of the equipment used. In our setup the silane/ammonia flow ratio that resulted in *x* = 1.49 iwa set to 45 sccm/550 sccm and a further reduction of silane flow resulted in unacceptable run-to-run non-repeatability. This is a practical limitation and equipment may be feasible for depositing stable materials at a very small ammonia/silane ratio.

The second constraint is more fundamental and concerns the diminishing potential for additional gains at *x* > 1.49. The increase in the optical bandgap of SiN_x_ with *x* saturates at about *E*_g_~5.5 eV, which would imply a spectral position of the onset of the associated spectral shift of the slope of exponentially increasing *k* in the range 260–280 nm. This limitation would make applications for the detection of, for instance, BTEX gases with gas-specific absorption spectra in the 240–280 nm band unlikely. Moreover, as shown in Figure 3, any shift in application window towards the shorter-wavelength UV enabled by a reduced *k* should be assessed relative to the associated reduction in index *n*, and thus reduced refraction index contrast between *n*_H_ and *n*_L_. Consequently, increasing the nitride composition beyond *x*~1.5 is unlikely to bring significant benefits in DBR design beyond those presented here.

The main achievements of the work presented are the validation of the suitability of nitride-rich SiN_x_ as an optical material with a relatively high index of refraction and low optical loss in near-UV, the establishment of an accurate optical database for SiN_x_, and an accurate design procedure for a SiN_x_/SiO_2_-based FPI with favorable optical properties in near-UV. The performance of the FPI with *m* = 15 and λ_o_ = 330 nm can be directly extracted from Table 2 and result for a SiN_1.49_-based design in a peak transmission of 64%, with FSR_15_ = 20 nm and FWHM_15_ = 2 nm. Future research includes the use of this material in practical applications.

## Figures and Tables

**Table 1:** Thickness of layers used in the final designs.

Symbol	Si_3_N_4_	SiN_1.39_	SiN_1.49_	SiO_2_
*n*_H_(λ_o_) ^1^	2.213	2.136	2.021	
*n*_L_(λ_o_)				1.492
*t*_H_ [nm]	37.3	38.6	40.8	
*t*_L_ [nm]				55.2
*t*_res_(*m* = 14) ^2^				1200 (1216)
*t*_res_(*m* = 15) ^2^				1320 (1327)
*t*_res_(*m* = 16) ^2^				1440 (1438)

^1^ Note that the number of digits used for presenting *n* indicates the resolution used in the calculation. The measurement inaccuracy is about 1% and the number of significant digits is limited to three. ^2^ In brackets is the result from calculation with the effect of the phase penetration depth on resonance included, while the rounded values are used as the nominal values for simulations and layer deposition.

**Table 2:** Characteristics of the two SiN_1.49_-based designs (also shown in Figure 12b and Figure 13b).

Design	Measured			Simulated			Mode	
	λ_peak_ [nm]	*T*_peak_	λ_FWHM_ [nm]	λ_peak_ [nm]	*T*_peak_	λ_FWHM_ [nm]	*FSR_m_* [nm]	*m*_der._ ^1^
*t*_res_ = 1192 nm, 300–320 nm band	304.1	0.595	2.6	304.3	0.507	2.2		
320–340 nm band	324.9	0.641	2.2	324.7	0.593	1.5	20.8	14.62
340–360 nm band	348.7	0.779	3.5	348.9	0.843	2.4	23.8	13.65
*t*_res_ = 1440 nm, 300–320 nm band	312.2	0.565	2.0	312.1	0.565	1.4		
320–340 nm band	330.8	0.631	1.9	330.6	0.639	1.4	18.6	16.77
340–360 nm band	351.8	0.772	3.1	351.8	0.862	2.1	21.0	15.75

^1^ Calculation of *m*_der_ is based on Equation (4): *m*_der_ = (λ_peak,m_−λ_peak,m+1_)/*FSR*_m_.

## Data Availability

Data can be made available by contacting the corresponding author.
